# Is endoanal, introital or transperineal ultrasound diagnosis of sphincter defects more strongly associated with anal incontinence?

**DOI:** 10.1007/s00192-020-04274-9

**Published:** 2020-03-20

**Authors:** Ingrid Volløyhaug, Annika Taithongchai, Linda Arendsen, Isabelle van Gruting, Abdul H. Sultan, Ranee Thakar

**Affiliations:** 1 Department of Clinical and Molecular Medicine, Norwegian University of Science and Technology, Trondheim, Norway; 2 Department of Obstetrics and Gynecology, Trondheim University Hospital, P.O. Box 3250, Sluppen, 7006 Trondheim, Norway; 3 Obstetrics and Gynaecology, Croydon University Hospital, Croydon, UK

**Keywords:** Anal sphincter, Faecal incontinence, Anal incontinence, Ultrasound, Anal pressure, Obstetric anal sphincter injury

## Abstract

**Introduction and hypothesis:**

Our aim was to explore the association between anal incontinence (AI) and persistent anal sphincter defects diagnosed with 3D endoanal (EAUS), introital (IUS) and transperineal ultrasound (TPUS) in women after obstetric anal sphincter injury (OASI) and study the association between sphincter defects and anal pressure.

**Methods:**

We carried out a cross-sectional study of 250 women with OASI recruited during the period 2013–2015. They were examined 6–12 weeks postpartum or in a subsequent pregnancy with 3D EAUS, IUS and TPUS and measurement of anal pressure. Prevalence of urgency/solid/liquid AI or flatal AI and anal pressure were compared in women with a defect and those with an intact sphincter (diagnosed off-line) using Chi-squared and Mann–Whitney *U* test.

**Results:**

At a mean of 23.6 (SD 30.1) months after OASI, more women with defect than those with intact sphincters on EAUS had AI; urgency/solid/liquid AI vs external defect: 36% vs 13% and flatal AI vs internal defect: 27% vs 13%, *p* < 0.05. On TPUS, more women with defect sphincters had flatal AI: 32% vs 13%, *p* = 0.03. No difference was found on IUS. Difference between defect and intact sphincters on EAUS, IUS and TPUS respectively was found for mean [SD] maximum anal resting pressure (48 [13] vs 55 [14] mmHg; 48 [12] vs 56 [13] mmHg; 50 [13] vs 54 [14] mmHg) and squeeze incremental pressure (33 [17] vs 49 [28] mmHg; 37 [23] vs 50 [28] mmHg; 36 [18] vs 50 [30] mmHg; *p* < 0.01).

**Conclusions:**

Endoanal ultrasound had the strongest association with AI symptoms 2 years after OASI. Sphincter defects detected using all ultrasound methods were associated with lower anal pressure.

## Introduction

Obstetric anal sphincter injury (OASI) is reported to occur after 0.5–20% of vaginal deliveries; with prevalence varying between hospitals, countries and different modes of delivery [1, 2]. OASI is a risk factor for anal incontinence (AI) later in life, with up to 60% developing symptoms of AI over time [3–5]. Correct diagnosis and proper suturing of the anal sphincters immediately after delivery is important to restore anatomy and function [6–8]. Diagnosis of residual defects of the external (EAS) and internal anal sphincter (IAS) and measurement of anal pressure, along with AI symptoms, can be used to predict the risk of deterioration of sphincter function after a subsequent vaginal delivery to counsel women regarding mode of delivery [9–11].

Endoanal ultrasound (EAUS) is the reference standard for imaging of the anal sphincters and diagnosis of sphincter defects, and correlates with symptoms and histological diagnosis [12, 13]. Alternatively, the anal sphincters can be examined with introital (IUS) and transperineal ultrasound (TPUS) using an endovaginal or abdominal probe [13–16]. In contrast to endoanal probes, endovaginal and abdominal probes are available in most obstetric and gynaecological units, and the examination is associated with less discomfort [17]. Previous studies have reported high inter-rater reliability [15] and strong correlation between AI and diagnosis of EAS defects on TPUS among urogynaecological patients [18, 19].

The EAS and IAS are not only morphologically different but also have different functions; the IAS mainly contributes to the resting pressure and the EAS is responsible for the voluntary squeeze [20]. Therefore, the presenting symptoms of the patient depend on the muscle that is injured [20]. Two previous studies found that injury diagnosed with EAUS had a stronger correlation with total AI symptom scores than IUS and TPUS [16, 17], but more detailed analyses of EAS and IAS defects in association with urgency, solid, liquid and flatal incontinence have not been carried out. Some studies have examined the association between anal canal pressures, AI and sphincter defects [4, 6, 11, 21, 22]. These studies included only up to 50 women with OASIs, but we found no studies assessing anal pressure in a larger population of women with healed and persisting sphincter defects.

Our primary aim was to explore the association between AI, including urgency, solid, liquid and flatal incontinence, and defects of the EAS and IAS diagnosed using 3D EAUS, IUS and TPUS in women who sustained OASI. Second, we aimed to study the association between persistent anal sphincter defects and anal pressure and establish the correlation between AI and anal pressure.

## Materials and methods

This was a cross-sectional study including 250 consecutive women who had sustained OASI and had primary sphincter repair immediately after delivery. They were referred to the perineal clinic at Croydon University Hospital, UK, between October 2013 and August 2015. This is a tertiary referral centre where all women sustaining OASI are examined 6 to 12 weeks postpartum and during any subsequent pregnancy to plan the mode of delivery. Advice regarding mode of delivery in a subsequent pregnancy is provided after EAUS examination of the anal sphincters, assessment of anal canal pressures and review of AI symptoms. Women 18 years or older who could read and understand English were eligible for study participation. The study was approved by the National Research Ethics Service South East London Committee, REC number 13/LO/0232 and was registered at clinicaltrials.gov NCT 02655900. All study participants gave written informed consent. The current study is a sub-analysis of a study assessing test accuracy of IUS and TPUS compared with EAUS for diagnosis of anal sphincter defects, and a power calculation of this parent study showed that 250 women were needed [17].

Symptoms of AI were assessed using the validated modified St Mark’s Incontinence Score (SMIS), ranging from 0 (no symptoms) to 24 (severe incontinence) [20, 23]. We then calculated the proportion of women with SMIS more than 0, and in addition the proportion with any faecal urgency, solid or liquid AI and any flatal AI, as these symptoms correlate with EAS and IAS function respectively [20]. Anal manometry was performed using the validated Stryker 295 air-filled pressure manometer system [21]. Maximum anal pressure was measured at rest and squeeze and the increment from rest to squeeze was calculated. Pelvic floor muscle strength was assessed by palpation using the Modified Oxford Scale, ranging from 0 (no discernible contraction) to 5 (strong contraction) [24].

**Fig. 1:**
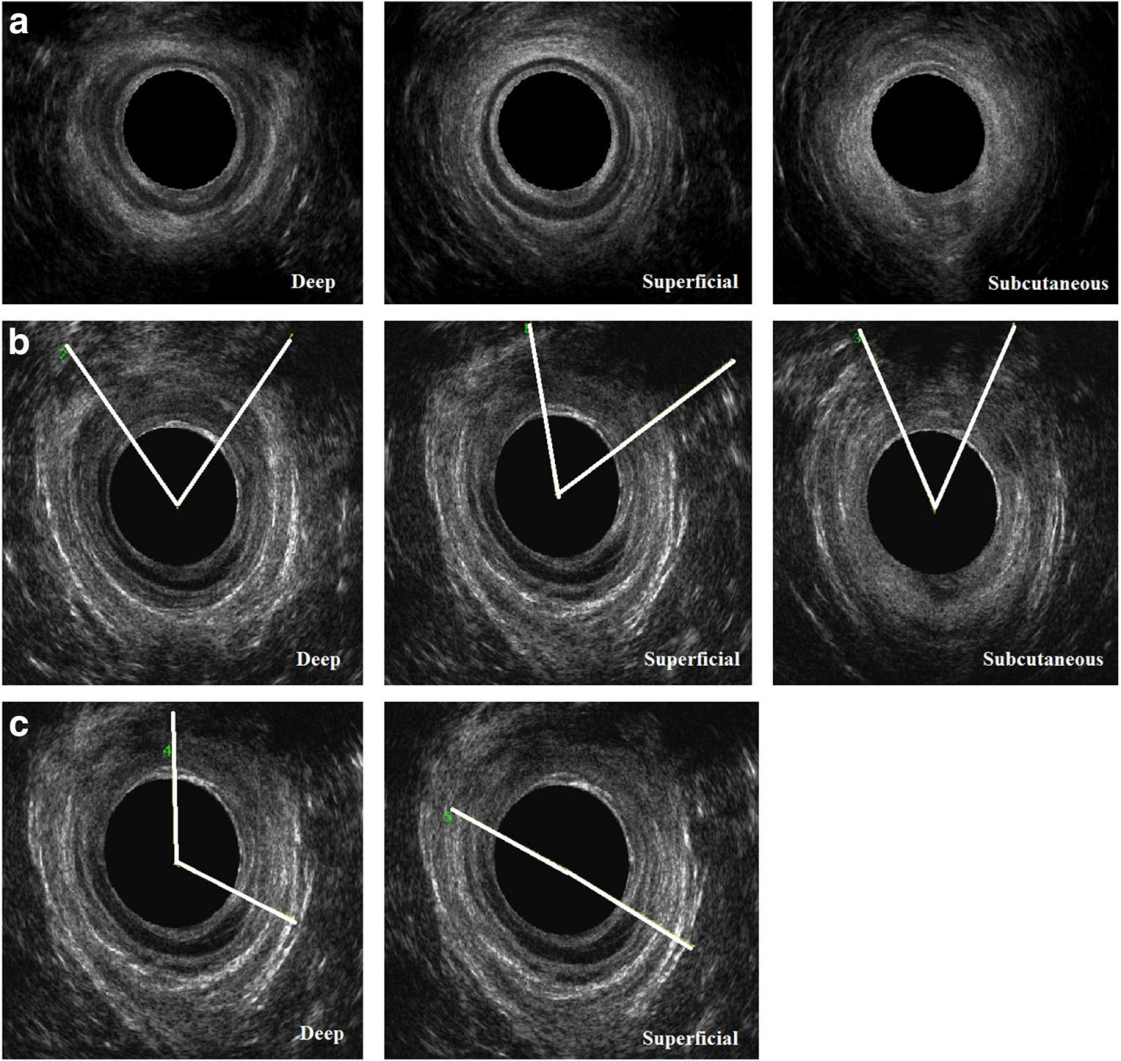
Endoanal ultrasound of the anal sphincters: slices at the deep (*left column*), superficial (*middle column*) and subcutaneous levels (*right column*). **a** Intact sphincters. **b** Defect in the external anal sphincter (hypoechoic ring) indicated by the angles. **c** Defect in the internal anal sphincter (hyperechoic ring) indicated by the angles

**Fig. 2:**
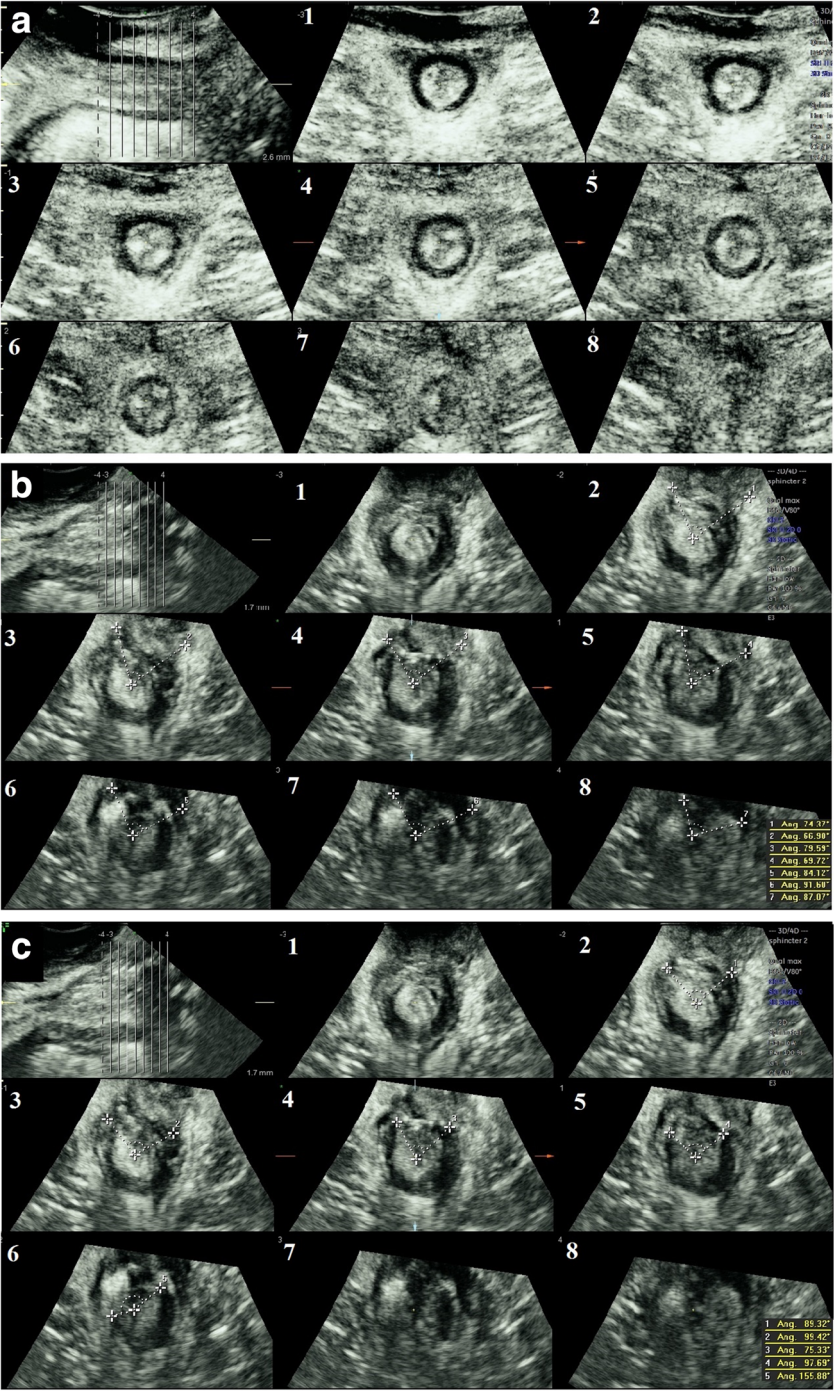
Introital/transperineal ultrasound of the anal sphincters. Tomographic ultrasound imaging demonstrating the puborectalis level (*slice 1*), the deep level (*slice 2*) of the internal and external anal sphincters, the superficial level (*slices 3–6*) of the internal and external anal sphincters and the subcutaneous level (*slices 7–8*) of the external anal sphincter. **a** Intact sphincters. **b** Defect in the external anal sphincter indicated with *dotted lines*. **c** Defect in the internal anal sphincter indicated with *dotted lines*

All women underwent an ultrasound examination performed by an investigator experienced in the imaging of the anal sphincters (IvG) after 8 months of rigorous training by the senior investigator (RT). EAUS was performed with the women lying in the left lateral position using either the BK Medical Pro-focus 2202 or BK Flex-focus 500 scanner (Gentofte, Denmark), fitted with a 12–16 MHz (type 2050) anorectal transducer (focal point up to 20 mm and focal range 5–45 mm, 360º acquisition angle). The women were examined in the supine position with knees and hips semi-flexed, using the GE Voluson i scanner (Zipf, Austria) with a 3D/4D 5- to 9-MHz endovaginal probe (IUS) at the posterior fourchette and a 3D/4D 4– to 8.5-MHz curved array abdominal probe (TPUS) placed transversely on the perineum, both with an acquisition angle of 85º. Three ultrasound volumes of each modality were stored for off-line analysis, and the best volume of each modality was used to assess sphincter integrity. Analyses of the EAUS volumes were performed using the BK3D viewer programme (version 7.0) and of the IUS and TPUS volumes using GE 4D view software (versions 10.2 and 14.0, GE Medical Systems). The EAUS volumes were analysed at the deep, superficial (middle) and subcutaneous levels of the EAS, and the IAS at the deep and superficial (middle) levels, as described previously (Fig. 1) [7] . The IUS and TPUS volumes were analysed using tomographic ultrasound imaging (TUI; Fig. 2) [17]. The IAS and EAS were analysed using the same TUI, where the interval between the slices was adjusted according to the individual length of the EAS. The first slice was at the puborectalis level, slice 2 at the deep level, slices 3–6 at the superficial level and slices 7 and 8 at the subcutaneous level of the EAS [17]. The IAS was visualised at the proximal and superficial levels (slices 2–6). On EAUS a significant defect of the EAS or IAS was diagnosed if a ≥ 30º defect was present at ≥1 level of the sphincter complex (Fig. 1b, c) [10]. On IUS and TPUS a significant EAS defect was diagnosed if a ≥30º defect was present in ≥3 out of 7 slices (Fig. 2b), and a significant IAS defect if a ≥30º defect was present in ≥2 out of 5 slices (Fig. 2c). These cut-offs have the highest sensitivity and specificity when tested against EAUS as the reference standard [17].

Imaging analysis was performed by three independent investigators who were blinded to the women’s symptoms, obstetric history and clinical findings. All three investigators analysed a series of 30 volumes of each imaging modality. An intraclass correlation coefficient (ICC; two-way-random, mean of three raters, absolute agreement model) was calculated for the Norderval score for each modality; grading the length, depth and radial extension of the EAS and IAS defect, score 0 being no defect and score 7 denoting maximum defect [17, 25]. The remaining 220 volumes of each modality were analysed by one rater: EAUS by AT, IUS by IV and TPUS by LA. The integrity of the levator ani muscle was assessed using tomographic ultrasound imaging at the level of the plane of minimal hiatal dimensions and 2.5 and 5 mm above this plane [26], and any uni- or bilateral major injury was registered.

Both the mean and median Norderval score and the proportion of women with a significant EAS and IAS defect were calculated for each ultrasound modality. The Chi-squared test was used to compare the proportion of women with faecal urgency/solid/liquid AI and intact and defect EAS and those with any flatal AI with intact and defect IAS. The effect of possible confounders on AI was tested; ongoing pregnancy, parity ≥2, significant levator ani muscle injury (uni- or bilateral) and grade of tear ≥3c were tested using Mann–Whitney *U* test. Age at examination, body mass index (BMI), pelvic floor muscle strength and time since index delivery were tested using Spearman’s rank correlation. Variables associated with the SMIS were entered into a multiple logistic regression model. The association of anal pressures with significant EAS and IAS defects for each ultrasound modality was assessed with the Mann-Whitney *U* test. Finally, the correlation between anal pressures and symptoms was tested with Spearman’s rank correlation. Statistical analysis was performed with IBM SPSS statistics version 23 software (IBM SPSS, Armonk, NY, USA). A *p* value < 0.05 was considered statistically significant for all analyses.

## Results

In total, 250 women were examined at a mean 23.6 (SD 30.1) months after the index (OASI) delivery. Mean (SD) age at examination was 31.5 (4.5) years and BMI 25.3 (4.7) kg/m^2^. A perineal tear grade 3a or 3b was diagnosed in 194 out of 223 (87%), and 29 out of 223 (13%) had a tear grade 3c or 4. Some women were referrals from other hospitals, and therefore 27 had missing data for OASI grade. Major levator injury was found in 73 out of 248 (29%) of the women (artefact affecting the levator in two volumes). In total, 65 out of 248 (26%) had more than one vaginal delivery and 88 out of 250 (35%) were pregnant at examination. Ethnicity was Caucasian 116 (46%), Indian 55 (22%), other Asian 35 (14%), black 27 (11%) and 17 (7%) of mixed or unknown ethnicity. At off-line analysis, 2 EAUS, 2 IUS and 4 TPUS volumes were missing. In some volumes there was an artefact, or the entire length of the sphincter was not captured, making it possible to evaluate 223 volumes of the EAS and 241 of the IAS in all 7 (EAS) and 5 (IAS) slices on IUS. On TPUS, it was possible to evaluate 227 volumes of the EAS and 238 of the IAS.

The ICC for the Norderval score indicated good interrater reliability: EAUS 0.83 (95% CI 0.70–0.92), IUS 0.76 (95% CI 0.57–0.88) and TPUS 0.86 (95% CI 0.74–0.93) [17]. The Norderval score and proportion of women with significant EAS and IAS defects are presented in Table 1. Only 61 women (24.4%) had AI. The mean (SD) SMIS was 1.4 (3.1) and the median SMIS was 0 (range 0–16) for the whole population. The mean SMIS was 5.6 (3.8) and the median SMIS was 4 (1–16) among women with SMIS >0. The proportion of women with urgency/solid/liquid AI and flatal AI in those with significant EAS and IAS defects and intact sphincters (including non-significant defects) detected on EAUS, IUS and TPUS is presented in Table 2. On EAUS, a significant difference between women with intact and defect sphincters was found for urgency/solid/liquid AI and flatal AI. For TPUS, this difference was only significant for flatal AI, and for IUS, no significant difference was found. Higher age at examination correlated positively with SMIS, *r*_*s =*_ 0.17, *p* = 0.01, but ongoing pregnancy, parity ≥2, levator ani muscle injury, pelvic floor muscle strength, grade of tear ≥3c, BMI, and time since index delivery were not associated with AI. Multiple logistic regression analysis adjusting for age at examination did not change the results.

**Table 1:** Norderval score [25] and proportion of significant defects of the external anal sphincter (EAS) and internal anal sphincter (IAS) diagnosed on endoanal, introital and transperineal ultrasound in 250 women with previous obstetric anal sphincter injury

Ultrasound modality	Norderval score Mean (SD) Median (range)	Significant EAS defect *n/N*%	Significant IAS defect *n/N*%	Significant IAS or EAS defect *n/N*%	Significant IAS and EAS defect *n/N*%
Endoanal *n* = 248	1.2 (2.0) 0 (0–7)	73/248 (29.4%)	34/248 (13.7%)	79/248 (31.9%)	28/248 (11.3%)
Introital *n* = 248	1.8 (1.9) 2 (0–7)	80/223 (35.9%)	52/241 (21.6%)	103/226 (45.6%)	29/222 (13.1%)
Transperineal *n* = 246	1.1 (1.5) 0 (0–7)	96/227 (42.3%)	19/238 (8.0%)	98/229 (42.8%)	17/227 (7.5%)

**Table 2:** Proportion with anal incontinence (AI) for women with intact (including non-significant defects^a^) and defect external anal sphincter (EAS) and internal anal sphincter (IAS) diagnosed with endoanal, introital^b^ and transperineal^c^ ultrasound

	Intact sphincter *n/N*%	Defect sphincter *n/N*%	Chi-squared test, *p*
Any faecal urgency, solid or liquid AI versus EAS defect			
Endoanal ultrasound	23/175 (13.1)	26/73 (35.6)	<0.01
Introital ultrasound	24/143 (16.8)	19/80 (23.8)	0.21
Transperineal ultrasound	20/131 (16.0)	25/96 (26.0)	0.06
Any flatal AI versus IAS defect			
Endoanal ultrasound	27/214 (12.6)	9/34 (26.5)	0.03
Introital ultrasound	27/189 (14.3)	7/52 (13.5)	0.90
Transperineal ultrasound	28/219 (12.8)	6/19 (31.6)	0.03

^a^<2 out of 5 slices with an IAS defect and <3 out of 7 with an EAS defect

^b^On introital ultrasound, it was possible to evaluate 223 volumes of the EAS and 241 of the IAS in all 7 (EAS) and 5 (IAS) slices

^c^On transperineal ultrasound, it was possible to evaluate 227 volumes of the EAS and 238 of the IAS in all slices

A highly significant difference in anal pressure at rest and squeeze increment was found between women with intact and those with defect sphincters detected on all three ultrasound techniques (Table 3). The correlation between the total SMIS and anal pressure was *r*_*s*_ = −0.06, *p* = 0.36 for resting pressure and *r*_*s*_ = −0.27, *p* < 0.01 for squeeze incremental pressure.

**Table 3:** Anal pressure for women with intact (including non-significant defects) and significant external and/or internal anal sphincter defects diagnosed using endoanal, introital and transperineal ultrasound

Pressures, mmHg	Both sphincters intact Mean (SD) Median (range)	Any significant sphincter defect Mean (SD) Median (range)	Mann–Whitney *U* test, *p*
Endoanal ultrasound			
Rest	55 (14) 55 (15–100)	48 (13) 46 (24–96)	<0.001
Squeeze increment	49 (28) 43 (3–161)	33 (17) 31 (2–70)	<0.001
Introital ultrasound			
Rest	56 (13) 55 (29–100)	48 (12) 48 (24–96)	<0.001
Squeeze increment	50 (28) 45 (10–161)	37 (23) 35 (2–133)	<0.001
Transperineal ultrasound			
Rest	54 (14) 55 (30–100)	50 (13) 50 (24–99)	0.016
Squeeze increment	50 (30) 43 (10–161)	36 (18) 33 (2–96)	0.002

## Discussion

In this study we found that an EAS defect correlated with urgency, solid or liquid AI, and that an IAS defect correlated with flatal AI on EAUS, in keeping with the function of these muscles [20]. IUS did not discriminate between symptomatic and asymptomatic women. On TPUS, the diagnosis of IAS defects was associated with flatal AI. Anal pressures at rest and incremental rise with squeeze were significantly lower for women with sphincter defects diagnosed using all ultrasound modalities. A lower incremental rise with squeeze correlated with a higher total modified St. Mark’s score.

One previous study used EAUS, IUS and TPUS to assess the association between persisting sphincter defects and AI symptoms in 55 women, and, similar to our study, they found an association between persisting defects and AI only with EAUS [16]. Our study is hitherto the largest study, including 250 women diagnosed with OASI at delivery. Women with different ethnicities were included, increasing the external validity. We used the modified SMIS, which is a validated and widely used questionnaire, to assess women’s AI symptoms [23] and found similar results to the previous study using the Wexner score [16]. Separate analysis of EAS and IAS defects in relation to urgency/solid/liquid AI and flatal AI provided more detailed information in the present study.

Most previous studies using TPUS have included women with a wider age range and longer time since delivery [18, 19, 27]. Any difference in symptoms between women with intact and injured sphincters could be easier to demonstrate after a longer time since the OASI. This may explain why these other studies found a difference in symptoms between intact and injured sphincters that was not reproduced in the present study. Furthermore, these studies included nulliparous women and women delivered only by caesarean section, in addition to women with OASI [18, 19, 27, 28]. Ultrasound diagnosis of a sphincter that has never been injured or repaired is easier than diagnosis of an injured and sutured sphincter, and therefore in these other studies, the distinction between intact and injured sphincters may have been easier with TPUS too. In the present study, only women with diagnosed and repaired OASI were included, in whom the scarring and disruption of the perineal tissues made the distinction between a repaired intact sphincter and one with a residual defect more difficult. This could explain why symptoms were not significantly different in women with and without sphincter defects on 3D IUS (EAS and IAS) and TPUS (EAS). Valsky et al. found no sign of tear and repair in 40% of women originally diagnosed with OASI [28]. It is unlikely that real tears heal so well that the scar was not recognised on ultrasound. A more plausible explanation, as shown by Sioutis et al. [29], is that there has been overdiagnosis of OASIs, mistaking the torn superficial transverse muscle for the anal sphincter, and consequently increasing the association with symptoms.

Different criteria for the diagnosis of significant sphincter defects have been applied in previous studies [16, 18, 19, 27, 28]. In contrast to the definition used by Guzmán Rojas et al. for TUI [18], we included two slices visualising the subcutaneous part of the EAS. Inclusion of slices covering the subcutaneous part of the EAS increased the sensitivity and specificity of IUS and TPUS compared with EAUS in our study population, and we therefore argue that this method is valid [17].

In a previous study, resting pressure below 40 mmHg or incremental rise in pressure less than 20 mmHg were considered abnormal [11]. In our study, the mean pressures were higher even for women with sphincter defects. The difference in resting pressure between women with intact and defect sphincters was similar to another previous study, but squeeze pressures were higher [21], supporting the hypothesis that women in our study may have had  too high anal pressures to become symptomatic. Some studies have found that levator ani muscle injury and pelvic floor muscle strength are associated with AI [30]. These factors had no impact on AI in the present study, suggesting that the integrity of the anal sphincters might be the most important factor for AI. It is, however, possible that an injured and weakened pelvic floor could contribute to AI over time.

The mean follow-up time after delivery was 24 months and only a few women were examined up to 10 years after the index delivery. Women had a mean age of 31.5 years at examination. This was a relatively short follow-up, as most symptoms related to a persisting sphincter defect may manifest as the women become older [5]. Time since index delivery did not correlate with symptoms, suggesting that within this relatively short period, the presence of a persisting defect on EAUS was more predictive of symptoms than the time factor. Our results implicate EAUS as the ultrasound modality that is better correlated with AI symptoms and should therefore be the preferred examination method in women who have sustained OASIs.

Endoanal ultrasound is part of the standard care at the perineal clinic at Croydon University Hospital; therefore, one weakness is that the examiner who acquired ultrasound volumes was potentially better trained in EAUS than in the other methods. Clinical counselling of the women was based on the EAUS examination, and the high quality of the ultrasound volumes was ascertained before storage of the volumes, making the off-line analysis of EAUS volumes easier. IUS and TPUS volumes were acquired at the same consultation, but analysis was only performed off-line, meaning that the quality of these volumes was not evaluated at the time of examination. The BK device used for EAUS provides ultrasound volumes of superior quality compared with the Voluson i system used for IUS and TPUS. Therefore, this may disfavour IUS and TPUS in comparison with EAUS. Furthermore, in spite of high ICC for the three examiners for all ultrasound modalities, it is possible that when single examiners proceeded to evaluate one modality, discordant interpretation occurred, which was dependent on the examiner and not on the ultrasound modality.

## Conclusions

Endoanal ultrasound diagnosis of sphincter defects appears to be the method with the strongest association with AI symptoms in women who have sustained OASI examined on average 2 years after delivery. IUS did not distinguish between symptomatic and asymptomatic women and only IAS defects diagnosed on TPUS were associated with higher symptom scores. Nevertheless, it is possible that the diagnostic accuracy of these methods improves with higher resolution ultrasound machines and dedicated training of examiners. Sphincter defects detected using all three ultrasound methods were associated with lower anal pressures, which in turn were associated with higher symptom scores. A longer-term follow-up would be needed to investigate the association between sphincter defects diagnosed on IUS and TPUS and symptoms over time.
